# Giving Consent to the Ineffable

**DOI:** 10.1007/s12152-024-09545-6

**Published:** 2024-02-15

**Authors:** Daniel Villiger

**Affiliations:** Institute of Philosophy, University of Zurich, Zollikerstrasse 117, 8008 Zurich, Switzerland

**Keywords:** Psychedelic-assisted therapy, Ethics, Informed consent, Transformative experience, Rationality, Testimony

## Abstract

A psychedelic renaissance is currently taking place in mental healthcare. The number of psychedelic-assisted therapy trials is growing steadily, and some countries already grant psychiatrists special permission to use psychedelics in non-research contexts under certain conditions. These clinical advances must be accompanied by ethical inquiry. One pressing ethical question involves whether patients can even give informed consent to psychedelic-assisted therapy: the treatment’s transformative nature seems to block its assessment, suggesting that patients are unable to understand what undergoing psychedelic-assisted therapy actually means for them and whether it aligns with their values. The present paper argues that patients often have sufficient knowledge to give informed consent because they know that they want to change their negative status quo and that psychedelic-assisted therapy offers an effective way to do so. Accordingly, patients can understand what the transformative nature of psychedelic-assisted therapy means for them and a make a value-aligned choice even if they are unable to anticipate the manifestation of a psychedelic experience.

## Introduction

Psychedelics are back on stage in mental healthcare [1]. Over the past decade, a growing number of studies have examined the therapeutic effects of classic serotonergic psychedelics, including psilocybin, lysergic acid diethylamide (LSD), and N,N-dimethyltryptamine (DMT).^1^ So far, the results are promising, suggesting effectiveness in treating cancer-related depression and anxiety [6–10], (treatment-resistant) depression [6, 11–17], and alcohol and smoking cessation [18–23]. But even though psychedelics have been ascribed great potential to have a major impact on mental healthcare in the coming decades, caution is still in order. Most of the current results stem from phase 1 studies, with only two phase 2 studies being published so far [13, 17]. Due to that, some researchers warn against creating inflated expectations of psychedelics’ therapeutic power as this leads to a psychedelic hype bubble that sooner or later bursts [24]. Nevertheless, one positive aspect of the current psychedelic excitement is that research on the topic accelerates: as of 2022, there were 30 ongoing phase 2 studies on psilocybin-assisted therapy and in January 2023, the first phase 3 study has started [25, 26]. Therefore, the next few years will show how much of the presumed therapeutic potential of psychedelics will prove to be true.

Next to the ongoing empirical examination of psychedelics’ therapeutic effects, the ethics of *psychedelic-assisted therapy* (PAT) has become an increasingly discussed topic [27–38]. Such discussions are urgently needed as the number of psychedelic trials is constantly increasing and, under certain conditions, countries such as Switzerland, Australia, or Canada already grant psychiatrists special permission to use PAT in non-research contexts. Moreover, in the first psychedelic wave in the 1950s and 1960s, researchers have often paid too little attention to psychedelics’ ethical implications, resulting in unethical practices that likely contributed to psychedelics’ controversial standing [39, 40]. To avoid the exploitation of patients and convince the public of psychedelics’ benefits in mental healthcare, it is essential that clinical psychedelic research gets accompanied by ethical inquiry.

One aspect of the ethical discussion concerns the informed consent process for PAT. Referring to the reasonable person standard in medical ethics, Smith and Sisti [36] persuasively argue that the informed consent process should be more comprehensive for PAT than what may be typical for other psychiatric medications. This is due to some of PAT’s unique features such as the ineffability of a psychedelic experience [41], the potential to shift patients’ values and personality [42–44], the possibility of therapeutic touch [45], and the presence of (rare) mental health risks [12, 46]. Consequently, these aspects should be disclosed and discussed with the patient during the informed consent process. But is having such information sufficient for the patient to be able to give informed consent?

A current hot topic in analytic philosophy casts doubt that it is. With her book *Transformative Experience*, L. A. Paul [47] questions whether a certain type of decisions can be made rationally, namely transformative decisions. Several authors have described a psychedelic experience as being transformative [48–52]. If that is true, patients may not be able to rationally choose (or decline) to undergo PAT. In turn, if we assume that aspects essential to rational choice are also essential to informed consent (e.g., having an understanding of an outcome’s consequences [cf. 52]), patients may not be able to give informed consent to PAT – even in the enhanced version proposed by Smith and Sisti [36]. The present paper thoroughly analyzes this line of thought and, in this way, comprehensively applies the transformative experience literature on PAT: it examines the transformative nature of PAT, how it is said to affect or not affect the ability to give informed consent, and why informed consent to PAT is possible despite the treatment’s transformative nature.^2^ In doing so, the paper contributes to the literature on psychedelic ethics by showing that: (1) Smith and Sisti’s [36] arguments for why the transformative nature of PAT does not pose a special problem for informed consent are not persuasive; and (2) contrary to Jacobs’s [52] position, informed consent for PAT can nonetheless be possible.

The remainder of the paper is structured as follows: Section 2 discusses transformative experiences, the challenges they pose to rational choice, and how this relates to psychedelics. Section 3 analyzes why PAT’s transformative nature poses a special problem for informed consent. Section 4 presents the paper’s account which demonstrates that giving informed consent to PAT can still be possible. Section 5 discusses practical implications for the informed consent process.

## The Transformative Experience Framework and How Psychedelic Experiences Fit into It

In her highly influential book *Transformative Experience*, Paul [47] argues that rational choice is impeded when at least one of the available options involves a transformative experience. At this, an option’s transformative nature can be twofold: First, if we have not experienced an outcome before, doing so transforms us *epistemically* as only by experiencing the outcome we learn how it is to experience it. Second, experiencing the outcome can transform us *personally*, meaning that it radically changes our point of view. From a decision-theoretic perspective, this goes along with a change of core preferences. Both types of transformation block the assessment of the option’s expected value: the epistemic transformation prevents us from knowing how it would be like if the outcome were to occur; and the possibility of a personal transformation complicates the decision situation even more as we no longer know on which preferences our decision should be based.

Paul [47] uses the term transformative experience for experiences that are both epistemically and personally transformative. The literature’s most prominent example of such an experience is becoming a parent [47, 53–62]. Only by becoming a parent, an agent gets to know how it is to be a parent. The outcome cannot be anticipated beforehand, at least not in a reliable manner. Moreover, becoming a parent can change the agent’s preferences: things that were of great importance before being a parent such as socializing or pursuing a career might no longer be of that much importance after becoming a parent. Due to the transformative nature of becoming a parent, an agent cannot assess its expected value which in turn blocks the ranking of available options. Expected value maximization is impeded since the agent does not know which of the available options maximizes (expected) value and cannot choose accordingly.

At this point, it is important to mention that an outcome’s transformative nature only veils part of its value, namely its subjective value (sometimes also called the experiential value or the phenomenal value). The subjective value is experientially grounded and refers to how it is to *live* an outcome [63]. Paul [47] argues that we assess an outcome’s subjective value by running a mental simulation of the outcome from which we can then derive its subjective value. Of course, the problem is that we cannot run a (reliable) mental simulation of a transformative experience as we do not have sufficient knowledge to do so. Contrary to this, we can assess an outcome’s non-subjective value as doing so does not depend on our ability to mentally simulate the outcome. But according to Paul [47], this is only of partial help since the non-subjective value alone is not decisive in the decisions she is interested in (she calls them first-personal choices).

Several authors have already referred to Paul’s concept of transformative experiences when writing about psychedelic experiences [e.g., 48–52]. This reference fits well. Undergoing a psychedelic experience for the first time is certainly epistemically transformative. It comes with profound changes in perception and mood, including phenomena such as ego dissolution [64], near-death-like experiences [65], paranoid and delusional thinking [66], and altered time perception [67]. These experiences are often described as being ineffable and inapprehensible before having them. Next to the epistemic transformation, a psychedelic experience can also be personally transformative as it bears the potential to shift one’s values and personality [42–44]. In fact, many first-time users of psychedelics say that the experience was one of the most significant ones in their life [7, 68]. Therefore, taking psychedelics can lead to both an epistemic and a personal transformation, making it a transformative experience in the Paulian sense. Ultimately, as far as we currently know, the value of taking psychedelics as part of PAT seems to be closely tied to experiencing the phenomenal aspects of a psychedelic trip [cf. 38]. If that is true, the subjective value of taking psychedelics should at least be co-decisive when deciding whether to undergo a psychedelic experience in the context of PAT. However, this would also imply that we cannot rationally choose to start PAT since we cannot mentally simulate the psychedelic experience and, consequently, not assess its (expected) subjective value.^3^ Therefore, patients who think about starting PAT are confronted with a transformative decision as they are unable to assess the (expected) value of undergoing a psychedelic session. Does this also affect their ability to give informed consent to PAT?

## Does Psychedelic-Assisted Therapy Pose Special Problems for Informed Consent?

The concept of informed consent is typically argued to be based on five elements: capacity, disclosure, understanding, voluntariness, and consent [69, 70]. PAT poses a challenge to the element of understanding [cf. 52]: Can we have an adequate understanding of PAT even though we cannot mentally simulate its outcome, do not know whether and how it will personally transform us, and are unable to assess its (expected) value?^4^

Smith and Sisti [36] believe that we can. In their enhanced informed consent proposal for PAT, the authors take the transformative nature of psychedelics into account. They suggest giving patients disclosure information about the experience itself, as for example: “You may feel a sense that you have lost yourself, that everything is somehow connected, or that all is one.” (p. 811) Such information refers to psychedelics’ epistemically transformative nature. Additionally, the authors also suggest giving patients disclosure information about potential long-term changes such as: “You may feel a greater sense of extroversion and openness to new experiences and ideas.” (p. 811) Such information refers to psychedelics’ personally transformative nature.

Despite these efforts to inform patients about PAT’s transformative nature, Smith and Sisti [36] already anticipated the objection that their proposal does not yet create a situation where informed consent becomes possible. At this, they refer to bioethicists such as Savulescu [75] who, much like Paul, emphasize the importance of mental simulation in choosing an option rationally and autonomously (which they see as relevant to informed consent). Smith and Sisti [36] agree that if informed consent to PAT required the ability to fully mentally simulate a psychedelic experience, such consent would not be possible. However, they doubt that being unable to fully mentally simulate an outcome undermines patients’ ability to give consent. To support their point, they write:

After all, we regularly accept consent to various activities that cannot be fully imagined—including beginning new relationships, getting married, starting a job and moving. Likewise, we take consent to traditional psychotherapy as authoritative despite effects on personality and worldview that subjects cannot fully appreciate before therapy. (p. 812)

Therefore, a partial mental simulation is sufficient to give informed consent, which is something that patients are capable of doing with regard to PAT.

I agree with Smith and Sisti [36] that giving informed consent does not require the ability to fully mentally simulate the relevant outcomes but that a partial mental simulation can be sufficient. Moreover, I also agree with the authors that the examples they use demonstrate that giving consent is possible in the context of transformative experiences. However, I reject the argument that, from the perspective of mental simulation, PAT is similar to the examples they use and therefore cannot pose a problem for informed consent either.

There are two main differences between Smith and Sisti’s [36] examples of transformative experiences where giving consent is possible and psychedelic experiences. First, most of their examples are what Carel and Kidd [76] call cumulative transformative experiences, meaning that not a singular event leads to a transformation but the cumulation of several events. For example, when starting a new relationship, there is not the one event that transforms you, but the transformation happens gradually as you get to know each other and come closer. Similarly, in psychotherapy, the transformational process is usually very slow and takes months if not years. In the context of such cumulative transformative experiences, agents can reevaluate their given consent after each of the experience’s subparts and abort the transformation if desired. And while starting a new job or moving are typically not cumulative transformative experiences, these outcomes can be rather easily altered or even reversed. For example, if you do not like the city you moved to, you can move to another city, including the one you came from. Or if you do not like your new job, you can quit and look for another job, including your previous job. In contrast to these examples by Smith and Sisti (2021), the transformation associated with a psychedelic experience result from a single event, and it is difficult to alter (let alone reverse) its consequences because of its personally transformative power. If, as it is often case, the psychedelic experience belongs to one of your most meaningful experiences, it will have a major impact on you. You cannot just shake off such an experience and leave it behind you – something that is likely possible in the job or moving example – since the experience will inevitably change your point of view. Ultimately, your control over a psychedelic experience is limited: a major advice is to surrender to the experience because fighting back is typically counter-productive [cf. 37]. In addition, psychedelics are believed to make you more suggestible and sensitive to context – also for some time after the psychedelic trip [39, 77–82]. Both of these aspects may reduce your control over how you integrate the experience into your life.

Second, the experiential novelty of Smith and Sisti’s [36] examples is rather limited. Of course, starting psychotherapy, a new relationship, or a new job comes with new experiences that cannot be fully anticipated. Nonetheless, by means of prior experiences and third-personal information, we can get a grasp of what these transformative experiences will likely be like. This is different in the case of a psychedelic experience. For example, it is not possible to have any comprehension of what it is like when your ego dissolves before experiencing it. Similarly, you cannot understand the potential significance of your psychedelic experience before having it. Smith and Sisti [36] seem to be aware of that. As part of their suggested disclosure information, they have the following sentence that refers to the communication with higher powers or the understanding of deeper realities:

Those who have experienced this often find it difficult to convey to others exactly what they experienced. Hence, we cannot tell you exactly what this is like, and you may have trouble understanding it before you experience it yourself. (p. 811)

But how should a person reading this disclosure information then be able to even partially simulate the possible mystical experience that comes with psychedelics? This seems not possible which suggests that our imaginative capacities to anticipate a psychedelic experience are much more limited compared to other transformative experiences. More generally, psychedelics put us in an experiential state that differs immensely from usual experiential states. Due to the large epistemic gap between these experiential states, we cannot anticipate a psychedelic experience through prior experience, and third-personal information is not very helpful here either. This explains why psychedelic experiences are often described as being ineffable: the words associated with usual experiential states are insufficient for describing psychedelic experiential states since the states’ experiential characteristics have too little overlap.

Because of the two reasons given above – (1) already a single psychedelic session can lead to major irreversible transformations and (2) a psychedelic experience comes with massive experiential novelties – giving consent to PAT is not as straightforward as in Smith and Sisti’s [36] examples. But what about other common examples of transformative experiences? If we look at the most prominent one in the literature – becoming a parent – we realize that it is more appropriate: like taking psychedelics, becoming a parent is a singular, irreversible transformative experience that comes with great experiential novelties. Since we do not question that two persons can give valid consent to becoming parents, it could be argued that the same must be true for taking psychedelics.

I do not want to settle for this line of argument for three reasons: First, although becoming a parent also comes with new experiential states, these states can still be better anticipated than psychedelic experiential states. You might be able to grasp how it will be to unconditionally love someone because of your other relationships; you might be able to grasp what family life will be like because of your own birth family and/or the family of friends and siblings; you might be able to grasp what long-term sleep deprivation will feel like because of prior instances of sleep deprivation; you might be able to grasp what it will be like to take care of someone because you took care of others before (e.g., your siblings, the children of siblings and friends, a pet). We do not have the same range of clues for grasping a psychedelic experience.

Second, the clinician-patient relationship likely comes with special obligations – obligations that are not present in a partner relationship. This is because the clinician-patient relationship is “an asymmetrical, professionalized relationship between a fiduciary and a vulnerable person, governed by the duty of care” [52]. This may provide a reason for more demanding consent requirements in the medical context than in the private context, and an argument for why consent to become a parent is possible, but consent to initiate PAT is not. In fact, we find several authors in the medical ethics literature who argue that for some interventions (e.g., surrogacy, experimental surgical clinical trials, or treatments that can lead to serious illness) informed consent may not be possible due to their transformative nature [e.g., 83–85]. Consequently, while the idea that one can generally give consent to actions is not questioned in the private context, this is not the case in the medical context (and thus it is not entirely exceptional to cast doubt on the possibility of giving informed consent to a treatment such as PAT).^5^ Again, one reason for this may be different consent requirements in these two contexts.

Third, even though we generally do not question that two persons can give valid consent to becoming parents, this is not proof that two persons can truly give valid consent to becoming parents. Put differently, maybe we should question the premise that two persons can give valid consent to becoming parents in the first place. At least, Paul [47] argues in this direction when discussing informed consent in her book: if we assume that the justification of consent is rooted in our ability to understand our values and preferences regarding different possible outcomes, we might not be able to give consent to becoming parents because of its transformative nature. Overall, for these three reasons, the argument that since we do not question that two persons can give valid consent to become parents, we should also not question that a person can give informed consent to PAT is not persuasive enough.

One obvious way out is to reject the idea that giving informed consent requires the agent to understand their values and preferences regarding different possible outcomes. For example, it could be argued that as long as patients are informed about the transformative potential of PAT and they comprehend that PAT is transformative, they have a sufficient understanding of PAT and can give informed consent. According to Jacobs [52], however, such an understanding of informed consent is misguided. He argues that one function of informed consent is to promote value-aligned decision-making, which he more or less equates with rational decision-making. Because of that, he shares Paul’s concerns about the possibility of informed consent in the presence of transformative treatments: since rational decision-making is not possible under such circumstances, informed consent is not possible either. In the words of Jacobs: “[S]ince the relevant information about PAP [psychedelic-assisted psychotherapy] is epistemically inaccessible at the point of deciding whether to commence with treatment, a patient cannot provide informed consent to the transformative facets of PAP as we standardly deploy the term[.]” (p. 7).

Now, the aim of the present paper is not to discuss whether Jacobs’ understanding of informed consent is correct: it remains agnostic here. Instead, the remainder of the paper aims to show that informed consent to PAT is possible *even if* it requires an adequate understanding of (1) one’s values and preferences regarding different possible outcomes and of (2) what the transformation means for oneself.^6^

## Gaining an Understanding of Psychedelic-Assisted Therapy

When discussing the challenges that transformative choices pose to informed consent in the medical context, Paul [47] mentions the example of a congenitally blind adult who thinks about retinal surgery. She describes the adult as follows:

[He] has built his life around his blindness, choosing a career (he is a saxophone player, whose soulful music reflects his lived experience and his highly trained auditory capacities) and a way of living and understanding the world through touch and sound, a way of living that is deeply tied to his blindness. (p. 159)

Should this person undergo retinal surgery? Paul is skeptical that decision theory can provide an answer to this question. She refers to potential higher-order properties that seeing shares with other experiences of which the saxophonist is familiar with. Based on these higher-order properties, a partial mental simulation of a retinal surgery’s outcomes can be run. Still, the knowledge of such higher-order properties only enables abstract approximations and cannot reveal what it is like to see. Besides, gaining a new sense is likely to affect the experience of the pre-existing senses as well which could have a major impact on his passion for playing the saxophone and the way he lives his life more generally. Anticipating such personal transformations and understanding their implications is very difficult even if the saxophonist has some knowledge of the experience’s higher-order properties. Because of these epistemic inaccessibilities, the saxophonist does not know whether undergoing retinal surgery provides more expected value than not doing so. In turn, this casts doubt on whether the saxophonist has a good enough grasp of the possible outcomes to give informed consent.

If we turn to an exemplary case of a patient who thinks about starting PAT, the decision situation seems prima facie similar to that of the congenitally blind saxophonist who thinks about retinal surgery, suggesting a pessimistic outlook on the ability to give informed consent to PAT. But there are significant differences between the two cases. The way Paul describes the saxophonist, he does not seem to perceive his blindness as a major disability that substantially worsens his life. Instead, he appreciates his highly developed senses of hearing and touch whose enhancement is a consequence of his blindness. We could even say that his affinity for sound and music, which again is linked to his blindness, defines who he is. Therefore, the potential epistemic and personal transformations coming with a retinal surgery have something threatening as they might shake the very foundations of how he defines himself (and also wants to define himself).

In contrast, patients who think about starting PAT most likely perceive their mental illness as a major disability that substantially worsens their life. And even if there are some positive aspects of the mental illness (e.g., some kind of creative output dealing with the mental illness), the negative aspects tend to be much weightier. In the end, this is the very reason why people seek treatment, with PAT often becoming relevant when other treatments have already failed. Finally, the development of a mental illness comes with a personal transformation [cf. 85]. For instance, a person who falls into a depressive episode tends to lose vitality and motivation, become emotionally numb, worry and ruminate constantly, and see the world and the future through a negative filter [87]. We can say, then, that major depressive disorder radically changes one’s point of view during a depressive episode. But unlike in the case of the saxophonist, the depressed person does not largely embrace this point of view (and the circumstances that go along with it). Thus, the person longs for a personal transformation that changes their current point of view.^7^

So, the main difference between the saxophonist and a mentally ill person lies in their evaluation of the status quo. The saxophonist does not simply enjoy his life despite his blindness but has built up a way of living and a self-identity that is deeply tied to his blindness (and that he embraces). Gaining the ability to see through retinal surgery could make his life even better but it could also make it much worse by disturbing the grounds on which he has built his personal identity and his life (with the likelihood of these outcomes being epistemically inaccessible). Contrary to that, a mentally ill person seeking treatment deeply suffers from their condition and does not want it to substantially affect their point of view. Undergoing PAT could finally put an end to their suffering and allow them to adopt a point of view they can more fully embrace.^8^ Does the different evaluation of the status quo affect whether rational choice is possible?

The literature on transformative experiences suggests that it does and illustrates this with the example of gender transition.^9^ Clearly, gender transition often comes with both an epistemically and a personally transformative experience. Due to the transformative nature of gender transition, the question arises whether a trans person can rationally choose to undergo it. McKinnon [88] was the first to treat this question. In her analysis, she first simplifies the outcome space by reducing it to four outcomes: “not transition and happy”, “not transition and unhappy”, “transition and happy”, and “transition and unhappy.” She then argues that for many trans people, the probability of the outcome “not transition and happy” is extremely low, making the outcome effectively impossible. They know this fact from their own experience which constantly demonstrates that non-transition is associated with deep unhappiness; frequently, non-transition means depression, suicidal thoughts, and also suicide attempts. So, many trans people decide between non-transition and almost certainly living an unhappy life or transition and living a happy or unhappy life, with the probabilities for the latter two outcomes being unknown. In such a situation, choosing to undergo gender transition must be rational as it can hardly worsen the situation but at the same time has the potential to substantially improve it. While McKinnon [88] left open how decision theory can solve cases structured such as gender transition, I [60] later presented a respective account.

We can use a similar line of argument to demonstrate how choosing to start PAT can be rational. As the example of gender transition shows, the rationale behind undergoing a medical intervention does not only stem from the person’s ability to understand their values and preferences regarding the intervention’s possible outcomes. It also stems from the person’s ability to understand their values and preferences regarding the status quo. In the context of PAT, patients know that they do not want to continue with the status quo and that they need a personally transformative experience to escape it. Consequently, they are looking for ways to escape the status quo. As shown in the introduction, while the renaissance of psychedelic research is still in its early stages, there is more and more evidence indicating that PAT has therapeutic effects for several mental disorders. Put differently, PAT can lead to a personally transformative experience that results in an alleviation or even overcoming of symptoms. Therefore, PAT appears to be a promising method by which a patient can escape their negative status quo, giving the patient reasons to begin PAT.

But are these reasons sufficient for rationally choosing to start PAT? After all, it is possible that the outcome of PAT can be even worse than the status quo, which could potentially outweigh the expected benefits of PAT. In order to mitigate this worry, we need to approximately know four things: (1) the likelihood that PAT improves a patient’s overall situation; (2) the magnitude of the improvement; (3) the likelihood that PAT worsens a patient’s overall situation; and (4) the magnitude of the worsening. Regarding (1), looking at studies which were part of two recent systematic reviews of psychedelics’ therapeutic effects, the clinical response rate ranges from 45% to 100% [89, 90]. In addition, the meta-analysis of Haikazian et al. [91] finds a pooled response rate of 57%. Regarding (2), the remission rate in the studies analyzed by Andersen et al. [89] and Ko et al. [90] ranges from 20% to 58%, and Haikazian et al. [91] find a pooled remission rate of 45%. Besides, it has been repeatedly shown that 6–14 months after their last session, an average of 76% (range 58–94%) of participants rate their psychedelic experiences as among the most meaningful experiences of their entire lives [7, 38, 68, 92, 93]. Regarding (3), post-session negative symptoms occurred in 0.9% of 250 participants in studies at Johns Hopkins and, likewise, in 0.9% of 110 participants in studies at the Vollenweider laboratory in Switzerland [38]. Regarding (4), a systematic review on PAT including 43 studies concludes that “[n]o serious, long-term adverse events were reported directly attributable to drug ingestion” [94]. Other systematic reviews on adverse events come to the same conclusion [95, 96].

Even though these numbers are preliminary, the analysis of systematic reviews and meta-analyses on PAT point in a clear direction: while PAT’s potential for significant improvement is considerable, its potential for significant worsening due to adverse events is very low [89–91, 94–99]. This, combined with the patient’s desire to escape the status quo, provides sufficient reasons for the patient to begin PAT and is thus the rationale behind the decision. On the same basis, the patient is also able to give informed consent to PAT.

Four clarifications are in order. First, as can be seen, the presented account does not build on mental simulation as a way to understand what a psychedelic experience means – such accounts would be doomed to fail.^10^ Instead, it builds on what Paul [100] calls “reflective replacement.” Reflective replacement means that we replace our mental simulation with a scientific-based assessment and do so in a reflective manner. According to Paul, such replacement is legitimate if science can tell us with sufficient accuracy how positive or negative a transformative outcome’s subjective value will be. For example, an agent can rationally decline to take a dangerous drug such as heroin despite its transformative nature if science clearly indicates that taking the drug provides negative (long-term) subjective value. Now, the last paragraph has shown that patients can rationally choose to undergo PAT and thereby reflectively replace their mental simulation. In doing so, the process of reflective replacement allows the patient to understand on a non-experiential level what the transformative experience that comes with PAT means for them: PAT’s transformative nature is not just a (unwanted) side effect but constitutive for reaching a state where the patient has overcome or at least alleviated their symptoms. Importantly, the scientific literature relevant for the reflective replacement also includes testimony of people who have already undergone PAT [e.g., 41]. Often, such testimony illustrates how PAT’s transformative nature is linked to its therapeutic effects. Consulting such testimony can provide a valuable route to gain an understanding of what the transformative experience coming with PAT means for oneself.^11^

Second, it is important to note that the favorable risk–benefit profile of PAT, which is a prerequisite for being able to rationally choose it, only applies to the patient groups that have participated in PAT studies to date. For example, it does not apply to people with psychotic disorders, bipolar disorders, or positive family history regarding these disorders as they are excluded from PAT studies due to safety issues. In addition, the favorable risk–benefit profile of PAT is derived from small, highly controlled clinical trials that provide extensive support when needed. While it is prima facie unclear whether a larger sample size affects PAT’s risk–benefit profile, it is likely that a less controlled and supportive setting worsens the risk–benefit profile. For example, Barrett et al. [101] argue that while acute adverse psychological reactions to psychedelics are usually benign in controlled settings with proper screening, preparation, and support, they remain a safety concern in uncontrolled settings. The rates and severity of acute effects in more controlled vs. less controlled settings support this [102]. Now, having a challenging psychedelic experience is not per se seen as a negative thing, as such experiences are “often interpreted as meaningful in themselves and/or accompanied by positive emotions or feelings of growth” [38]. But then again, it is likely to be more difficult to put a positive spin on a challenging experience in a less supportive setting.

It can be expected that as PAT becomes more widely used, the setting becomes less controlled and supportive than in past and current clinical trials. To ensure that patients can also give informed consent to PAT in such settings, the effects of gradually relaxing the highly controlled setting of clinical trials must be studied. The gradual relaxation should still allow patients to rationally choose PAT based on previous evidence. At the very latest, further relaxation must be stopped when the risk–benefit profile turns out to be no longer favorable.

Third, even though consulting testimony does not enable patients to mentally simulate a psychedelic experience, it can be important in two other ways. First, as previously mentioned, it helps patients understand how PAT’s transformative nature and its therapeutic effects are intertwined with each other. Second, while consulting testimony does not enable patients to mentally simulate a psychedelic experience, it helps them classify aspects of their psychedelic experience when it is occurring. For example, when consulting testimony about ego dissolution, patients are unable to imagine what it is like. Nonetheless, knowing that psychedelics can lead to ego dissolution can be useful. This is because when a patient’s ego dissolves in a psychedelic session, they can classify their experience as an instance of ego dissolution and are not taken by complete surprise that something like that can happen. So, testimony (partly) prepares patients for the range of psychedelic experiences, while their experiential characteristics remain concealed until they actually manifest.

However, there might also be a risk that comes with consulting testimony. Several authors have described psychedelics as active super-placebos, meaning that they catalyze and amplify what is already there, including our expectations [29, 82, 103]. If patients’ expectations are significantly influenced by the testimonials that they consulted, these testimonials might ultimately affect the manifestation of their psychedelic experience [cf. 29]. This could lead to a trade-off between consulting testimony to prepare for the range of psychedelic experiences and having your very own psychedelic experience. While there is only little empirical research on this topic so far, future findings will be relevant from both a clinical and an ethical perspective.

Fourth, the presented account requires that patients perceive their mental illness as a major disability and suffer deeply from the status quo. As mentioned above, patients suffering from severe depression or anxiety can be expected to meet this requirement. However, psychedelics are not only used clinically to treat severe depression and anxiety but, for example, also for smoking cessation [e.g., 20]. Here it is questionable whether patients perceive their status quo in a markedly negative way and, if so, whether their nicotine addiction contributes significantly to this – prerequisites for a rational choice of PAT. This is not to say that patients suffering from nicotine addiction are per se unable to rationally choose PAT and therefore incapable of giving informed consent: there certainly are individuals with nicotine addiction who fulfil the requirements of the paper’s account. However, it suggests that it is appropriate to try other smoking cessation treatments whose consequences are easier to anticipate and understand first, and then perhaps PAT as a late-line treatment.^12^

There is empirical evidence to support the idea that informed consent for PAT may not typically be possible in the context of smoking cessation. A qualitative study on patients with nicotine addiction who underwent PAT found that, in retrospect, many consider smoking cessation to be one of the least important outcomes of the treatment [104]. This has two probable implications: First, their evaluation of the status quo was not substantially affected by their nicotine addiction. Otherwise, it is hard to explain why they did not consider quitting smoking as one of the top outcomes of the treatment. Second, they had little understanding what undergoing PAT would mean for them: when choosing PAT, they likely thought that the meaning of PAT for them would be to overcome their nicotine addiction, which turned out to be wrong. If these implications are true, then unlike patients suffering from severe depression or anxiety, these patients suffering from nicotine addiction could not rationally choose to undergo PAT and thus could not give informed consent. Therefore, for each mental disorder, it is necessary to assess whether patients are typically in a situation that allows them to rationally choose PAT and thus give informed consent.

## Practical Implications for the Informed Consent Process

The account presented in the last section shows that despite the transformative nature of PAT, informed consent is possible. What are the practical implications of the account for the informed consent process? First, as suggested by Smith and Sisti [36], it is important to provide patients information about possible epistemic as well as personal transformations coming with PAT. However, the intention behind doing so is not to enable patients to (partly) mentally simulate a psychedelic experience but to illustrate patients the range of possible psychedelic experiences (without expecting that they are able to understand these experiences). In addition, patients need to be told that despite the wide range of possible psychedelic experiences, the majority of those who have had a psychedelic experience derived a positive value from it, and only very few derived a negative value from it. At this, patients also need to be told that while these findings point in a clear direction, they are still preliminary.

Second, patients need to be informed about the connection between PAT’s transformative nature and its therapeutic effects. As part of their suggested disclosure information, Smith and Sisti [36] already have a sentence that goes into this direction: “The benefits of this intervention may be related to or depend on these effects of the experience and these changes to your personality.” (p. 811) But this alone does not sufficiently highlight the connection between PAT’s transformative potential and its therapeutic effects. For example, patients also need to be told that (1) to reach a state where they have overcome or at least alleviated their symptoms, they need to undergo a (personally) transformative experience that brings them there; that (2) there is increasing evidence showing that PAT can provide the desired transformative experience; and therefore that (3) it is important to understand that PAT’s transformative nature is not just a side effect but constitutive for its therapeutic effects (while this might not be true for every transformative aspect of the experience, it is certainly true for the transformative experience as a whole). In this connection, clinicians should show and discuss testimony of former patients which illustrates the role that PAT’s transformative nature has played in the process of overcoming or alleviating their symptoms.

Based on these two points, patients can develop an understanding of what the epistemic and personal transformations coming with PAT mean for them. Combined with Smith and Sisti’s [36] other suggested disclosure information, this puts patients in a position where they can give informed consent to PAT.

## Conclusion

The present paper has argued that patients often have sufficient knowledge about their values and preferences to give informed consent to PAT. This is because they know that they no longer want to continue with their negative status quo. In turn, escaping the status quo requires a respective transformative experience. As of yet, the evidence shows that PAT can provide such a transformative experience and also has a favorable risk–benefit profile, enabling a value-aligned decision (yet this is only true for the patient groups included in previous trials and for PAT performed in highly controlled settings). Thus, even though patients cannot anticipate the manifestation of a psychedelic experience, they can understand the function of its transformative nature and thereby what it means for them.

On a final note, the line of argument presented in this paper can be applied to transformative treatments more generally: when patients suffer deeply due to their physical/mental condition and need a transformative experience to escape their negative status quo, they can rationally choose a treatment with a favorable risk–benefit profile that is expected to provide such a transformation. Consequently, they are also in a position to give informed consent.

## Data Availability

Not applicable.

## Footnotes

1 Next to trials with classic serotonergic psychedelics, studies with 3,4-methylenedioxmethamphetamine (MDMA) have shown therapeutic effects for post-traumatic stress disorder [2, 3], including a phase 3 study [4], and for social anxiety in patients with autism [5]. However, this paper focuses on classic serotonergic psychedelics.

2 The analysis is limited to the legal medical application of PAT in research and non-research contexts.

3 This is also true if not every single psychedelic experience is transformative: already a non-negligible probability of a psychedelic experience being transformative makes it impossible to rationally choose to undergo such an experience. The reason for this is that we cannot know in advance whether the psychedelic experience will be transformative in our case.

4 Egerton and Capitelli-McMahon [71] also question the possibility of informed consent for transformative treatments, including PAT. They analyze decision-making capacity (DMC) in the context of transformative treatments and argue that in this context “the assumptions behind the principle of informed consent (that someone with DMC is in a position to weigh up the options available) fail” (p. 14). However, a widely accepted assumption about DMC is that any theory of it must result in most ordinary adults being considered capacitated most of the time [72–74]. The idea that there are medical decisions for which humans per se lack DMC seems odd. Even a variable standard of DMC would never set the bar for required skills so high that no one could meet them (including physicians and judges). Therefore, it is more persuasive that PAT’s transformative nature poses a challenge to the element of understanding than to the element of capacity in the informed consent framework.

5 An exception in the private context may be morally intolerable cases, where valid consent is sometimes considered insufficient for morally transformative consent [cf. 86].

6 Or in response to Egerton and Capitelli-McMahon [71] who, like Jacobs, argue that informed consent is not possible for PAT: the remainder of the paper aims to show that informed consent to PAT is possible *even if* it requires the patient to be “in a position to weigh up the options available” (p. 14).

7 To reach a point of view that is no longer significantly affected by one’s mental illness, one by definition needs a personal transformation. This is because every experience that substantially changes one’s point of view is personally transformative. So, the treatment of mental illness requires a personal transformation.

8 Note that the personal transformation may also be “restorative,” meaning that, e.g., a depressed person transforms to their pre-depressed self. In such a case, the personal transformation itself may not be epistemically transformative (while the psychedelic experience still is). However, before undergoing PAT, a patient does not know if it will lead to a personal transformation and, if so, how that transformation will unfold (i.e., in a restorative way or not). So, the general problem that PAT poses to informed consent remains.

9 It needs to be highlighted that gender dysphoria or gender incongruence is not a mental disorder. Still, if they are handled in the form of gender transition, medical intervention is required.

10 Of course, we can mentally simulate some aspects of the psychedelic experience as for example lying on a bed or wearing a blindfold. Maybe we might also be able to mentally simulate some optical effects of psychedelics if we watch a video of a psychedelic trip simulation beforehand (at best with virtual reality glasses). Nevertheless, these doable simulations do not concern the significant parts of the experience, namely the inner sensations and insights coming with a psychedelic experience.

11 While today’s preliminary evidence allows for reflective replacement, reflective replacement was likely not possible in many of the studies that established today’s evidence. This is because the evidence on the risk–benefit profile of PAT was much more limited at the time these studies were conducted. However, the aim of this paper is not to show that rationally choosing PAT and, in this way, giving informed consent to it has always been possible, but that it is possible now.

12 A possible rationale for using PAT as a late-line treatment for smoking cessation is as follows: It could be argued that if patients have tried several unsuccessful smoking cessation treatments and still have a strong desire to quit smoking, it indicates that they are suffering deeply from their condition. And since other treatments have already failed, it becomes rational to try PAT.
